# Effect of water storage on the color stability and surface roughness of resin composites with S-PRG fillers subjected to additional polymerization for semidirect use

**DOI:** 10.4317/jced.64101

**Published:** 2026-07-29

**Authors:** Lilian Almeida Nascimento Rabêlo, Waldemir Francisco Vieira-Junior, Cecilia Pedroso Turssi, Fabiana Mantovani Gomes França, Roberta Tarkany Basting

**Affiliations:** 1 Faculdade São Leopoldo Mandic, Divisão de Cariologia e Odontologia Restauradora, Campinas, Brazil

## Abstract

**Background:**

Additional polymerization in the semidirect technique improves the physicochemical properties of resin composites. However, the effects of long-term water storage on the color stability and surface roughness of resin composites containing S-PRG (Surface Pre-Reacted Glass) fillers remain unclear. This study evaluated the effect of autoclave polymerization on the color stability and surface roughness of a conventional nanohybrid resin composite and one containing S-PRG fillers after one year of water storage.

**Materials and Methods:**

Disc-shaped specimens of Forma (Ultradent) and Beautifil II LS (Shofu; containing S-PRG fillers) were prepared (N = 48). Half of the specimens (n = 12) underwent additional autoclave polymerization. Color parameters (L*, a*, b*, Eab, E00) and surface roughness (Ra) were measured at baseline (24 h) and after 12 months of storage in distilled water. Scanning electron microscopy (SEM) was performed. Data were analyzed using generalized linear mixed models (=0.05).

**Results:**

Color variation (Eab and E00) was significantly greater for the resin composite containing S-PRG than for the conventional nanohybrid resin composite (p<0.05), whereas the polymerization method did not affect color variation (p>0.05). Ra values did not differ according to resin composite type or polymerization method (>0.05); however, all groups exhibited a significant increase after storage (<0.05). SEM revealed no differences attributable to the polymerization method.

**Conclusions:**

Additional autoclave polymerization did not influence the color stability or surface roughness of resin composites used in the semidirect technique. However, the resin composite containing S-PRG fillers exhibited lower long-term color stability, highlighting the influence of filler composition on esthetic durability.

## Introduction

Resin composites are the material of choice for direct posterior restorations because of their esthetic properties, ability to bond to dental tissues through adhesive systems, reduced wear on healthy teeth, and lower cost compared with indirect restorative materials (1). To improve longevity and enhance physical and mechanical performance, resin composites have undergone continuous modifications, particularly in filler composition, morphology, and particle size, as well as in organic matrix composition (2). Nanohybrid resin composites were developed to improve polishability and gloss retention through the incorporation of nanometric fillers combined with larger filler particles with average sizes exceeding 100 nm, arranged as clusters or agglomerates (2). In addition, some resin composites have been formulated with bioactive S-PRG (Surface Pre-Reacted Glass) fillers, which consist of multifunctional alumino-fluoro-borosilicate glass particles ranging from 0.01 to 4.0 m (3). These bioactive fillers release several ions, including fluoride, borate, aluminum, sodium, silicate, and strontium, thereby contributing to bioactivity (3). In a two-year prospective controlled clinical trial, restorations containing S-PRG fillers demonstrated acceptable clinical performance and physical and mechanical properties comparable to those of conventional nanohybrid resin composites (4). However, resin composites with S-PRG fillers appear more susceptible to discoloration because of polymer matrix degradation and filler particle loss (5 - 7). In addition, the color stability of resin-based materials is influenced by intrinsic degradation mechanisms, including the degree of conversion and incomplete polymerization (8). A lower degree of conversion reduces crosslink density, thereby decreasing material hardness and increasing susceptibility to hydrolytic and enzymatic degradation, ultimately compromising physical and mechanical properties (9). Although direct resin composite restorations are widely used because of their lower cost and reduced chairside time, they may not always provide optimal occlusal anatomy, proximal contact, or long-term performance in extensive restorations. In such situations, semidirect resin composite restorations represent a valuable alternative because they combine the simplicity of direct techniques with some of the advantages of indirect procedures (10). In this approach, the restoration is fabricated extraorally on a cast or silicone mold and subsequently finished and cemented intraorally. A key benefit is the possibility of performing additional polymerization outside the oral environment using light and/or heat, thereby minimizing moisture contamination and increasing the degree of conversion (10 - 12). Consequently, semidirect restorations may improve clinical outcomes while remaining less expensive and less complex than fully indirect restorations. The semidirect technique further enhances polymerization through a post-curing process involving additional light, heat, and/or pressure, thereby improving the physical, mechanical, and esthetic properties of resin composites (10 , 11). Among the available post-curing methods, heat treatment (thermopolymerization) is an economical and practical approach (10 , 12) that can be performed using either a steam autoclave or a microwave oven (6 , 10 , 12). Previous studies have shown that this method improves microhardness, flexural strength, fracture toughness, wear resistance, and color stability (13). However, evidence regarding the effects of heat treatments on the surface roughness and color stability of S-PRG-containing resin composites intended for semidirect restorations remains limited, particularly after long-term water storage. Therefore, this study evaluated the surface roughness and color stability of resin composites containing S-PRG fillers after 12 months of water storage following additional autoclave polymerization for semidirect restorations. The null hypotheses were: H01) the surface roughness would not be affected by resin composite type, autoclave polymerization, or storage time; and H02) the color stability would not be affected by resin composite type, autoclave polymerization, or storage time.

## Materials and Methods

- Experimental Design This study evaluated 48 disc-shaped nanohybrid resin composite specimens randomly allocated to a 2 × 2 factorial design (n = 12) and analyzed using a split-plot design with time as the repeated factor. Sample size calculation was performed using G*Power (version 3.1.5; Heinrich Heine University Düsseldorf, Germany), based on F-tests for repeated-measures designs. The following parameters were adopted: = 0.05, statistical power (1 ) = 0.80, and a medium effect size (f = 0.50), according to classifications for comparable study designs (14). Although the minimum required sample size was 10 specimens per group, 12 specimens were included per group to account for potential losses. The factors under study were: (1) Resin composite type: conventional nanohybrid resin composite (Forma, Ultradent; control group without S-PRG fillers) versus nanohybrid resin composite containing S-PRG fillers (Beautifil II LS, Shofu). (2) Polymerization method: conventional photoactivation versus photoactivation followed by autoclave post-curing. (3) Time: baseline (initial) versus 12 months of storage. The response variables were surface roughness (Ra, m) and color parameters (L*, a*, and b*), including overall color change (Eab and E00). Material specifications are presented in Table 1.

Table 1

- Specimen Preparation Forty-eight nanohybrid resin composite discs were prepared using a Teflon mold (2 mm thick × 6 mm in diameter), with 12 specimens assigned randomly to each combination of resin composite type and polymerization method. The resin composite was inserted into the mold, which was positioned on a glass plate and covered with a polyester strip using an insertion spatula. A second polyester strip and glass plate were then placed over the material, and a 500 g weight was applied for 10 s to compact the material and eliminate entrapped air in the unpolymerized resin. The specimens were subsequently photoactivated using a Valo Corded LED curing unit (Ultradent Products, South Jordan, UT, USA) in standard mode, at a radiant emittance of 1000 mW/cm2 for 20 s. The base of each specimen was marked with a small indentation using a diamond bur for identification. - Additional Polymerization Method After photoactivation, specimens assigned to additional polymerization were autoclaved (Phoenix Lutenco, AV-30/2, 30 L; Indústria e Comércio de Equipamentos Científicos, Araraquara, SP, Brazil) at 127 °C for 6 min according to the protocol described by Grazioli et al. (10). The specimens were placed individually in surgical-grade paper envelopes before autoclaving. After autoclave treatment, they were allowed to cool at room temperature (23 ± 1 °C) and were subsequently stored in distilled water in Eppendorf tubes. - Finishing and Polishing The specimens were polished using a finishing and polishing system with coarse-, medium-, fine-, and superfine-grit discs (Sof-Lex Pop-On, 3M, Brazil), according to the manufacturer's instructions. Each specimen was polished for 20 s with each grit under a standardized force of 2 N, monitored using a balance. Between grit changes, the specimens were rinsed with distilled water for 10 s. Polishing discs were replaced after every five specimens. After polishing, the specimens were stored individually in Eppendorf tubes containing distilled water at 37 °C for 24 h, after which baseline measurements of surface roughness and color were obtained. - Surface Roughness Analysis Ra was evaluated at baseline and after 12 months of storage using a Surftest SJ-210 roughness meter (Mitutoyo Corporation, Kanagawa, Japan). Each specimen was measured at three locations, each passing through the geometric center of the disc. After each measurement, the specimen was rotated by 120°. The final Ra value corresponded to the mean of the three measurements. The measurement parameters were a static load of 4 mN, scanning speed of 0.05 mm/s, and cut-off value of 0.25 mm in sequential mode. - Color Analysis Color measurements were obtained using a digital spectrophotometer (VITA Easyshade, VITA Zahnfabrik) in a standardized light box with a white background at baseline and after 12 months of storage. Color parameters were analyzed according to the CIEL*a*b* system, in which L* represents lightness (0 = pure black, 100 = pure white), a* represents the green-red axis (+a* = red, a* = green), and b* represents the yellow-blue axis (+b* = yellow, b* = blue). The L*, a*, and b* values were calculated for each resin composite and time point. Total color change (Eab) was calculated using the following formula (13): Eab = (L*)2 + (a*)2 + (b*)2. The perceptibility and acceptability thresholds for Eab were set at 1.2 and 2.7, respectively (15). Color change was also evaluated using the CIEDE2000 (E00) formula, which incorporates hue (h) and chroma (C), as proposed by Sharma et al. (16). The perceptibility and acceptability thresholds for E00 were set at 0.8 and 1.8, respectively (15). - Storage The specimens were stored individually in Eppendorf tubes containing distilled water and maintained in an incubator at 37 ± 1 °C for 12 months. The distilled water was replaced weekly. - Scanning Electron Microscopy The surface micromorphology of two randomly selected specimens from each resin composite was evaluated after finishing and polishing and after one year of storage in distilled water. All surfaces were sputter-coated (estimated coating thickness: 200 Å) using a sputter coater (Emitech K450, Kent, UK). Photomicrographs were obtained at 3000× magnification using a high-resolution scanning electron microscope (Thermo Fisher Scientific, Model Quattro S, Thermo Scientific UltraDry, Brno, Czech Republic) operating at 20 kV. Qualitative surface analysis was performed to assess the presence of depressions and irregularities and to characterize filler morphology. - Statistical Analysis Statistical analyses were conducted using R software (17) at a significance level of 5% ( = 0.05). Initially, descriptive and exploratory analyses were conducted. Based on the characteristics of each variable, generalized linear mixed models for repeated measures over time were used for L*, a*, b*, and Ra, whereas generalized linear models were applied to Eab and E00. Changes in Ra were analyzed using the nonparametric Mann-Whitney test.

## Results

No significant differences in Ra were observed between the resin composites or between the polymerization methods (p > 0.05) (Table 2).

Table 2

However, Ra increased significantly in all groups after 12 months of storage (p < 0.05). The results for L*, a*, and b* are presented in Table 3.

Table 3

The L* parameter increased significantly after storage for the conventional nanohybrid resin composite (p < 0.05), whereas it decreased for the nanohybrid resin composite containing S-PRG fillers (p < 0.05). At baseline, the nanohybrid resin composite containing S-PRG fillers exhibited a significantly higher L* value than the conventional nanohybrid resin composite (p < 0.05) (Table 4). After storage, the a* parameter increased significantly for both resin composites (p < 0.05), with higher values observed for the nanohybrid resin composite containing S-PRG fillers than for the conventional nanohybrid resin composite (p < 0.05). Likewise, the b* parameter increased significantly for both resin composites after storage (p < 0.05). At baseline, the conventional nanohybrid resin composite exhibited lower b* values when photoactivation was combined with autoclave polymerization than when photoactivation alone was performed (p < 0.05). Conversely, the nanohybrid resin composite containing S-PRG fillers exhibited higher b* values when photoactivation was combined with autoclave polymerization (p < 0.05). Total color variation (Eab and E00) after storage was significantly greater for the nanohybrid resin composite containing S-PRG fillers than for the conventional nanohybrid resin composite (p < 0.05) (Table 4).

Table 4

**Figure 1:**
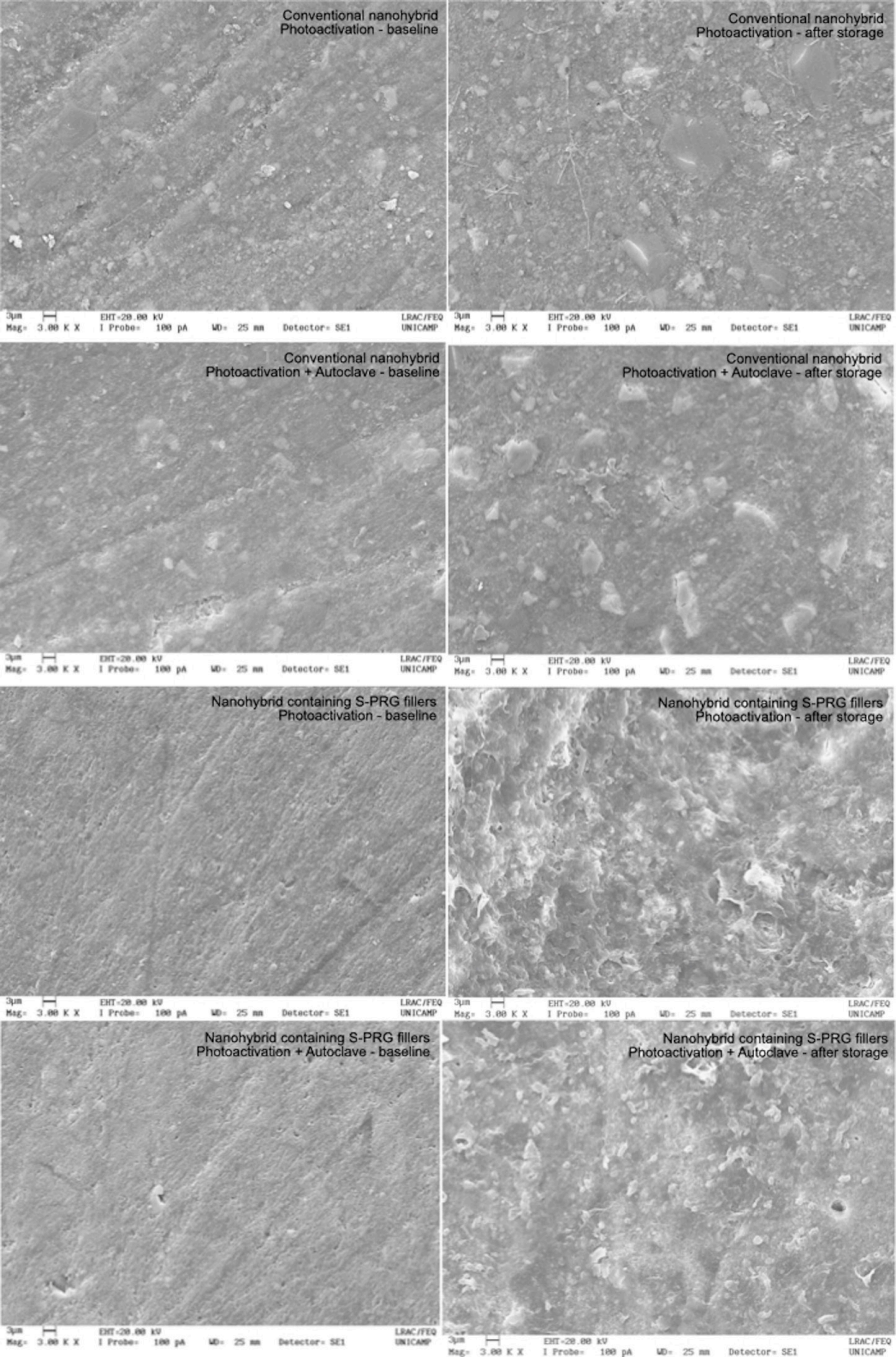
Micromorphology images (3000×) of conventional nanohybrid resin composites and nanohybrid resin composites containing S-PRG fillers, both photoactivated and photoactivated with autoclave polymerization, before (baseline) and after 12 months of storage in distilled water.

However, the polymerization method did not significantly affect color variation (p > 0.05). Micromorphological images (Figure 1) of the conventional nanohybrid resin composite showed fillers dispersed throughout the resin matrix, with irregular sizes and shapes in both the photoactivated and photoactivated + autoclave polymerization groups. Scratches produced during polishing were visible on the surface.

1

After one year of storage in distilled water, slight degradation of the resin matrix resulted in partial filler projection from the surface. No micromorphological differences were observed between the polymerization methods for the nanohybrid resin composite containing S-PRG fillers. Both groups exhibited relatively smooth surfaces, with only minor polishing scratches and no evident filler projections. After one year of storage, however, the resin matrix displayed an irregular surface morphology suggestive of water uptake in both the photoactivated and photoactivated + autoclave polymerization groups. The surface appeared heterogeneous, with visible depressions and cavities, suggesting surface degradation.

## Discussion

The surface roughness and color stability of restorative materials influence both the esthetic quality of restorations and their longevity (18). Greater stability of these properties may reduce biofilm adhesion and discoloration associated with degradation occurring in the oral cavity (19). Increased monomer conversion, which may be enhanced by autoclave heat treatment, has been proposed to improve surface smoothness and color stability (10 - 12). Although no significant differences in roughness were observed between the resin composites or between the polymerization methods, the first null hypothesis was rejected because surface roughness increased significantly after 12 months of storage. Autoclave polymerization employs high temperature and pressure to promote a more complete and homogeneous cure, thereby improving mechanical properties such as strength and dimensional stability (20). Although autoclave treatment may have increased monomer conversion (12), it did not significantly influence surface roughness in the present study. Moreover, roughness values remained below the 0.5 µm threshold for perceptible differences in surface texture (21) but exceeded the 0.2 µm threshold associated with increased biofilm retention (22). Consistent with these findings, micromorphological images of the nanohybrid resin composites showed no surface differences between the polymerization methods at baseline. These results highlight the importance of filler size, filler composition, and organic matrix composition in determining the surface roughness of resin composites (2 , 23). No significant differences in surface roughness were observed between the conventional nanohybrid resin composite (control group) and the resin composite containing S-PRG fillers. This similarity may be attributed to filler characteristics, including size, shape, volume, and composition (3). Since both materials are nanohybrids, they contain a combination of nanometric fillers and larger filler particles, which provide comparable physical and mechanical properties (2). However, differences in filler size appeared to influence other properties. A comparison of conventionally polymerized and autoclave-treated resin composites revealed similar roughness values despite differences in filler composition. This finding may be explained by the predominant influence of the polishing procedure on surface roughness (24). Polishing with aluminum oxide discs effectively removed surface irregularities and produced a smooth finish (7), thereby minimizing differences in surface roughness between the resin composite types. Conversely, surface roughness increased in all resin composites after one year of storage, regardless of autoclave polymerization. This increase may be associated with water immersion, which accelerates degradation of the organic matrix (25). Water exposure promotes silane hydrolysis and water sorption by the resin matrix, resulting in softening and progressive degradation (26). Consequently, microcracks may form and soluble components may leach from the material, contributing to increased surface roughness (27). In addition, water penetration into the resin matrix may induce surface plasticization, reducing material rigidity and altering surface smoothness (28). The resin composite containing S-PRG fillers appears to exhibit greater water sorption, which may explain its more pronounced surface changes over time and potentially affect its longevity. In contrast, the conventional resin composite may exhibit lower water sorption because of fewer hydrophilic functional groups in its resin matrix, thereby reducing surface plasticization (27). These findings are consistent with the micromorphological results obtained after 12 months and with the greater color changes observed in the resin composites containing S-PRG fillers. In addition to surface roughness, the clinical success of a restorative material depends on color stability. Because the color stability of the resin composite containing S-PRG fillers differed from that of the conventional resin composite, the second null hypothesis was rejected. Consistent with the present findings, previous studies (5 - 7 , 29) reported that nanohybrid resin composites containing S-PRG fillers are more susceptible to color change, possibly because their mechanism of action depends on water sorption for ion release. However, photoactivation combined with autoclave polymerization did not significantly affect the Eab and E00 color change values. These findings suggest that increased monomer conversion did not alter the organic matrix sufficiently to influence color stability. Although additional polymerization through heat and pressure has been reported to affect color stability (30), no such effect was observed in the present study. The results demonstrated significant differences in color stability between conventional nanohybrid resin composites and those containing S-PRG fillers after storage. The conventional nanohybrid resin composite exhibited a significant increase in L* (lightness) after storage, whereas the nanohybrid resin composite containing S-PRG fillers showed a significant decrease. This finding may be related to the specific characteristics of S-PRG fillers, which may interact differently with storage media than conventional resin composites (5). At baseline, the resin composite containing S-PRG fillers exhibited higher lightness, likely because of the nature and size of its fillers, which can reflect more light. However, the subsequent decrease in L* after storage suggests that S-PRG fillers may influence long-term color stability differently. Regarding the a* (red-green) and b* (yellow-blue) parameters, both resin composites exhibited significant changes after storage, with the resin composite containing S-PRG fillers presenting higher a* and b* values than the conventional resin composite. These findings indicate a shift toward more reddish and yellowish hues over time, which was more pronounced in the resin composite containing S-PRG fillers. Total color variation, measured by Eab and E00, was significantly greater for the resin composite containing S-PRG fillers, indicating a more noticeable color change that exceeded the perceptibility thresholds (15). One limitation of this in vitro study is that it was conducted under highly controlled conditions that do not fully replicate the clinical environment. The absence of saliva and erosive, abrasive, and mechanical challenges may limit the applicability of the findings. Future studies should simulate more clinically relevant conditions, evaluate additional storage periods, and investigate interactions between S-PRG fillers and the resin matrix, particularly regarding their effects on color stability and degradation mechanisms.

## Conclusions

Additional autoclave polymerization did not influence the color stability or surface roughness of the resin composites evaluated. However, resin composites containing S-PRG fillers exhibited greater color changes after one year of storage.

## Figures and Tables

**Table 1:** Resin composites, shades, and compositions of the resin composites used.

Resin composition	Manufacturer	Shade	Composition/Batch
Conventionalnanohybrid (Forma)	Ultradent(Indaiatuba, SP, Brazil)	A2E	Bis-GMA, TEGDMA, Bis-EMA, UDMA; zirconia-silica fillers and barium glass (64.8 vol%)Lot: D0LRB
Nanohybrid containing S-PRG fillers (Beautifil II LS)	Shofu Inc.(Kyoto, Japan)	A2E	Bis-GMA, triethylene glycol dimethyl ether, alumino-fluoro-borosilicate glass, aluminum oxide, DL-camphorquinone (68.6 vol%)Lot: 122275

Abbreviations: Bis-GMA = Bisphenol A-glycidyl methacrylate; Bis-EMA = Ethoxylated bisphenol dimethacrylate; TEGDMA: triethylene glycol dimethacrylate; UDMA = Urethane dimethacrylate.

**Table 2:** Mean (standard deviation) of surface roughness (Ra, μm) as a function of resin composite, polymerization method, and time.

Resin composite	Polymerization method	Baseline	After storage
ConventionalNanohybrid	Photoactivation	0.32 (0.08) Ba	0.48 (0.18) Aa
	Photoactivation + Autoclave	0.33 (0.06) Ba	0.40 (0.09) Aa
Nanohybrid with S-PRG fillers	Photoactivation	0.30 (0.08) Ba	0.41 (0.14) Aa
	Photoactivation + Autoclave	0.33 (0.12) Ba	0.51 (0.14) Aa

Different uppercase letters (horizontally) indicate significant differences between time points within the same polymerization method and resin composite. Different lowercase letters (vertically) indicate significant differences between polymerization methods within the same resin composite and time point (p ≤ 0.05). p-values (factor effects): resin composite: p = 0.9106; polymerization method: p = 0.5967; resin composite x polymerization method: p = 0.1357; time: p < 0.0001; resin composite x time: p = 0.4420; polymerization method x time: p = 0.7358; resin composite x polymerization method x time: p = 0.1703.

**Table 3:** Mean (standard deviation) of the L*, a*, and b* parameters as a function of resin composite, polymerization method, and time.

Variable	Resin composite	Polymerization method	Time	
			Baseline	After storage
L*	Conventional nanohybrid	Photoactivation	86.71 (1.76) a	87.39 (1.41) a
		Photoactivation + Autoclave	85.25 (1.79) a	85.99 (2.13) a
		Multiple comparisons	B	A
	Nanohybrid with S-PRG fillers	Photoactivation	*87.90 (1.14) a	86.90 (1.53) a
		Photoactivation + Autoclave	*87.76 (1.72) a	86.53 (1.52) a
		Multiple comparisons	A	B
Resin composite: p = 0.040; polymerization method: p = 0.062; resin x method interaction: p = 0.185; time: p = 0.161; resin x time interaction: p < 0.001; method x time interaction: p = 0.788; resin x method x time interaction: p = 0.550				
a*	Conventional nanohybrid	Photoactivation	2.71 (0.17) a	3.50 (0.17) a
		Photoactivation + Autoclave	2.83 (0.47) a	3.62 (0.44) a
		Multiple comparisons	B	A
	Nanohybrid with S-PRG fillers	Photoactivation	*4.65 (0.33) a	*5.64 (0.65) a
		Photoactivation + Autoclave	*4.63 (0.33) a	*5.74 (0.31) a
		Multiple comparisons	B	A
Resin composite: p < 0.0001; polymerization method: p = 0.3432; resin x method interaction: p = 0.4268; time: p < 0.0001;resin x time interaction: p < 0.0001; method x time interaction: p = 0.8334; resin x method x time interaction: p = 0.4205				
b*	Conventional nanohybrid	Photoactivation	26.54 (0.85) Ba	27.73 (0.84) Aa
		Photoactivation + Autoclave	25.21 (1.98) Bb	27.23 (1.13) Aa
	Nanohybrid with S-PRG fillers	Photoactivation	*21.53 (0.96) Bb	*24.86 (1.80) Aa
		Photoactivation + Autoclave	*22.79 (1.12) Ba	*25.35 (0.84) Aa
Resin composite: p < 0.0001; polymerization method: p = 0.7373; resin composite x method interaction: p = 0.0058; time: p < 0.0001; resin composite x time interaction: p < 0.0001; method x time interaction: p = 0.6503; resin x method x time interaction: p = 0.007				

(*) Indicates a significant difference from the conventional nanohybrid resin under the same polymerization method and time conditions (p ≤ 0.05). Different uppercase letters (horizontally) indicate significant differences between time points within the same polymerization method and resin composite. Different lowercase letters (vertically) indicate significant differences between polymerization methods within the same resin composite and time point (p ≤ 0.05).

**Table 4:** Mean (standard deviation) of ΔEab and ΔE00 as a function of resin composite and polymerization method.

	Polymerization method	Resin composite	
		Conventional nanohybrid	Nanohybrid with S-PRG fillers
ΔEab​	Photoactivation	1.81 (0.62) Ba	3.97 (1.19) Aa
	Photoactivation + Autoclave	2.40 (1.26) Ba	3.29 (1.04) Aa
p(resin)<0.001; p(method)=0.8839; p(resin x method)=0.0386			
ΔE00	Photoactivation	1.07 (0.24) Ba	2.06 (0.55) Aa
	Photoactivation + Autoclave	1.30 (0.58) Ba	1.80 (0.50) Aa
p-values: resin composite: p < 0.001; polymerization method: p = 0.9174; resin composite x polymerization method interaction: p = 0.0831			

Different uppercase letters (horizontally) and lowercase letters (vertically) indicate statistically significant differences (p ≤ 0.05).

## Data Availability

The datasets used and/or analyzed during the current study are available from the corresponding author.

## References

[1] Demarco FF, Collares K, Coelho-de-Souza FH, Correa MB, Cenci MS, Moraes RR, Opdam NJ (2015). Anterior composite restorations: A systematic review on long-term survival and reasons for failure. Dent Mater.

[2] Ferracane JL (2024). A historical perspective on dental composite restorative materials. J Funct Biomater.

[3] Imazato S, Nakatsuka T, Kitagawa H, Sasaki JI, Yamaguchi S, Ito S (2023). Multiple-ion releasing bioactive surface pre-reacted glass-ionomer (S-PRG) filler: Innovative technology for dental treatment and care. J Funct Biomater.

[4] Toz-Akalin T, Öztürk-Bozkurt F, Kusdemir M, Özsoy A, Yüzbaşıoğlu E, Özcan M (2023). Clinical evaluation of low-shrinkage bioactive material giomer versus nanohybrid resin composite restorations: A two-year prospective controlled clinical trial. Oper Dent.

[5] Pimentel ES, França FMG, Turssi CP, Basting RT, Vieira-Junior WF (2023). Effects of in vitro erosion on surface texture, microhardness, and color stability of resin composite with S-PRG fillers. Clin Oral Investig.

[6] Botrel BJ, Condi ML, Turssi CP, Gomes-França FM, Vieira-Junior WF, Basting RT (2025). Physical and mechanical properties of bulk-fill resin composites submitted to additional polymerization for use in semi-direct restorations. Oper Dent.

[7] Moreira ENT, Vieira-Junior WF, Turssi CP, França FMG, Basting RT (2025). Effect of polishing systems on the roughness, color, and staining of conventional and bulk-fill resin composites with and without S-PRG filler. Clin Oral Investig.

[8] Cadenaro M, Maravic T, Comba A, Mazzoni A, Fanfoni L, Hilton T (2019). The role of polymerization in adhesive dentistry. Dent Mater.

[9] Kowalska A, Sokolowski J, Bociong K (2021). The photoinitiators used in resin-based dental composites - A review and future perspectives. Polymers (Basel).

[10] Grazioli G, Francia A, Cuevas-Suárez CE, Zanchi CH, Moraes RR (2019). Simple and low-cost thermal treatments on direct resin composites for indirect use. Braz Dent.

[11] Carrillo-Cotto R, Silva AF, Isolan CP, Selayaran RPG, Selayaran M, Lima FG (2021). Effects of alternatively used thermal treatments on the mechanical and fracture behavior of dental resin composites with varying filler content. J Mech Behav Biomed Mater.

[12] Souza LS, Donato TR, Cerqueira GA, Cavalcanti AN, Mathias P (2021). Color stability of an artificially aged nanofilled resin composite post-cured with different techniques. J Dent Res Dent Clin Dent Prospects.

[13] (2004). CIE Technical Report: Colorimetry. CIE pub no 15.3.

[14] Zieliński G, Więckiewicz M (2025). New effect size and sample size guidelines in dentistry. Dent Med Probl.

[15] Paravina RD, Ghinea R, Herrera LJ, Bona AD, Igiel C, Linninger M (2015). Color difference thresholds in dentistry. J Esthet Restor Dent.

[16] Sharma G, Wu W, Dalal EN (2005). The CIEDE2000 color-difference formula: Implementation notes, supplementary test data, and mathematical observations. Color Res Appl.

[17] (2023). R Core Team. R: A language and environment for statistical computing.

[18] Aydın N, Topçu FT, Karaoğlanoğlu S, Oktay EA, Erdemir U (2021). Effect of finishing and polishing systems on the surface roughness and color change of resin composites. J Clin Exp Dent.

[19] Ferracane JL (2008). Buonocore Lecture. Placing dental composites--a stressful experience. Oper Dent.

[20] Braga RR, Ballester RY, Ferracane JL (2005). Factors involved in the development of polymerization contraction stress in resin-composites. Dent Mater.

[21] Jones CS, Billington RW, Pearson GJ (2004). The in vivo perception of roughness of restorations. Br Dent J.

[22] Bollen CM, Lambrechts P, Quirynen M (1997). Comparison of surface roughness of oral hard materials to the threshold surface roughness for bacterial plaque retention: A review of the literature. Dent Mater.

[23] Cunha LG, Alonso RCB, Souza-Júnior EJ, Neves AC, Sobrinho LC, Sinhoreti MA (2008). Influence of the curing method on the post-polymerization shrinkage stress of a composite resin. J Appl Oral Sci.

[24] Ruschel VC, Lauer F, Maia HP, Shibata S, Carvalho LD, Gré CP (2013). Effect of autoclaving on action of polishing systems on the surface roughness of a composite resin. J Appl Oral Sci.

[25] Ricci WA, Alfano P, Pamato S, Cruz CADS, Pereira JR (2019). Mechanical degradation of different classes of resin composites aged in water, air, and oil. Biomed Res Int.

[26] Malacarne J, Carvalho RM, de Goes MF, Svizero N, Pashley DH, Tay FR (2006). Water sorption/solubility of dental adhesive resins. Dent Mater.

[27] Ito S, Hashimoto M, Wadgaonkar B, Svizero N, Carvalho RM, Yiu C (2005). Effects of resin hydrophilicity on water sorption and changes in modulus of elasticity. Biomaterials.

[28] Huang W, Ren L, Cheng Y, Xu M, Luo W, Zhan D (2022). Evaluation of the Color Stability, Water Sorption, and Solubility of Current Resin Composites. Materials (Basel).

[29] Costa MP, Jacomine JC, Mosquim V, Santin DC, Zabeu GS, Agulhari MAS (2024). Analysis of color stability and degree of conversion of different types of resin composites. Braz Oral Res.

[30] Abdulmajeed AA, Suliman AA, Selivany BJ, Altitinchi A, Sulaiman TA (2022). Wear and color stability of preheated bulk-fill and conventional resin composites. Oper Dent.

